# Obesity paradox in cancer: Is bigger really better?

**DOI:** 10.1111/eva.12790

**Published:** 2019-04-24

**Authors:** Beata Ujvari, Camille Jacqueline, Dorothée Misse, Valentin Amar, Jay C. Fitzpatrick, Geordie Jennings, Christa Beckmann, Sophie Rome, Peter A. Biro, Robert Gatenby, Joel Brown, Luis Almeida, Frédéric Thomas

**Affiliations:** ^1^ Centre for Integrative Ecology, School of Life and Environmental Sciences Deakin University Deakin Victoria Australia; ^2^ School of Natural Sciences University of Tasmania Hobart Tasmania Australia; ^3^ Centre de Recherches Ecologiques et Evolutives sur le Cancer/Maladies Infectieuses et Vecteurs: Ecologie, Genéttique, Evolution, et Contrôle, CNRS UniversiteÂ de Montpellier Montpellier France; ^4^ Laboratoire Jacques‐Louis Lions Université Paris Descartes Paris France; ^5^ School of Science and Health Western Sydney University Parramatta New South Wales Australia; ^6^ CarMeN Laboratory (UMR INSERM 1060‐INRA 1397, INSA), Lyon‐Sud Faculty of Medicine University of Lyon Lyon France; ^7^ Department of Radiology H. Lee Moffitt Cancer Center & Research Institute Tampa Florida; ^8^ Laboratoire Jacques‐Louis Lions Sorbonne‐Université, CNRS, Université de Paris, Inria Paris France

**Keywords:** cancer, evolution, obesity, selective filter

## Abstract

While obesity is widely recognized as a risk factor for cancer, survival among patients with cancer is often higher for obese than for lean individuals. Several hypotheses have been proposed to explain this “obesity paradox,” but no consensus has yet emerged. Here, we propose a novel hypothesis to add to this emerging debate which suggests that lean healthy persons present conditions unfavorable to malignant transformation, due to powerful natural defenses, whereby only rare but aggressive neoplasms can emerge and develop. In contrast, obese persons present more favorable conditions for malignant transformation, because of several weight‐associated factors and less efficient natural defenses, leading to a larger quantity of neoplasms comprising both nonaggressive and aggressive ones to regularly emerge and progress. If our hypothesis is correct, testing would require the consideration of the raw quantity, not the relative frequency, of aggressive cancers in obese patients compared with lean ones. We also discuss the possibility that in obese persons, nonaggressive malignancies may prevent the subsequent progression of aggressive cancers through negative competitive interactions between tumors.

It is increasingly established that excess body fat is associated with higher cancer risk (Calle, Rodriguez, Walker‐Thurmond, & Thun, 2003; IARC, 2002; Marmot, Atinmo, Byers, & Chen, 2007). However, it is also commonly found that elevated body mass index (BMI; i.e., above 25 kg/m^2^, Arnold et al., 2016) is associated with improved survival among cancer patients compared to lean subjects (see Lennon, Sperrin, Badrick, & Renehan, 2016 and Wang et al., 2018 for recent reviews). This conflicting finding, termed the “obesity paradox,” is currently attracting increasing attention from oncologists and epidemiologists (Caan & Kroenke, 2017; Gonzalez, Pastore, Orlandi, & Heymsfield, 2014; Gupta, 2016; Mayeda & Glymour, 2017; Strulov Shachar & Williams, 2017). The “obesity paradox” has been demonstrated in patients with lymphoma, leukemia, colorectal, endometrial, thyroid, renal, and lung cancers (Caan & Kroenke, 2017; Zhang, Liu, Shao, & Zheng, 2017). For unclear reasons, the list of cancers that are apparently concerned by the obesity paradox is not necessarily the same as those cancers most commonly observed in obese persons: esophagus (adenocarcinoma only), colon and rectum, liver, gallbladder, pancreas, kidney, advanced prostate, post‐menopausal female breast, endometrium, and ovary cancers (Whiteman & Wilson, 2016).

Recent studies have suggested various phenomena that might explain the obesity paradox, but these have mainly focused on methodological problems including the crudeness of BMI as an obesity measure, confounds with other factors, detection bias, and reverse causality and collider‐stratification bias (Lennon et al., 2016; Park, Peterson, & Colditz, 2018). While these methodological limitations are possible, they are however unlikely to fully account for the observed findings (Caan & Kroenke, 2017).

In contrast to these explanations which suggest that the “obesity paradox” is an artifact of the data and its analysis and not a causal or functional link, we put forward here a mechanistic explanation for why this paradox might arise. We propose that the effects of obesity on oncogenic process dynamics coupled with its negative effect on the efficiency of natural defense mechanisms could wrongly suggest that obesity has a protective effect. Beside this possible misinterpretation, it remains also possible that in obese patients the same processes favor the early emergence of less aggressive tumors that subsequently prevent the development and growth of more aggressive ones.

While we often distinguish between people who have and do not have cancer, the reality is that virtually every individual develops small tumors in the body by the time they reach their sixth or seventh decade of life (Bissell & Hines, 2011; Folkman & Kalluri, 2004). The reasons why these lesions sometimes progress to clinical diseases are currently not fully understood, but at least partially depend on the efficiency of protective mechanisms to prevent the microscopic colonies of malignant cells from becoming lethal invasive tumors (Harris, Schiffman, & Boddy, 2017). There is also strong evidence indicating that an excess of fat negatively impacts immune functions and host defenses in obese individuals (Bandaru, Rajkumar, & Nappanveettil, 2013; Meckenstock & Therby, 2015; Milner & Beck, 2012), a process that is also likely to accelerate malignant progression (Bindea, Mlecnik, Fridman, Pagès, & Galon, 2010; Jacqueline, Biro, et al., 2016; Jacqueline, Bourfia, et al., 2016). Ducasse et al. (2015) also provided compelling evidence that cancer can be viewed as a particular manifestation of a quasi‐universal ecological process that is the proliferation of profiteering phenotypes in disturbed systems with unused resources. With the alteration of the internal ecosystem associated with obesity (e.g., increased levels of endogenous hormones, the contribution of abdominal obesity to gastroesophageal reflux and esophageal adenocarcinoma, inflammatory responses), together with abundant unused resources, a higher number and diversity of malignancies progress and become clinically evident (Nunez, Hursting, Yakar, Fowler, & Vinson, 2009; Price, Cavazos, Angel, Hursting, & deGraffenried, 2012). Recently Wang et al. (2018) demonstrated that obesity often results in increased immune aging, tumor progression, and PD‐1‐mediated T‐cell dysfunction partially driven by leptin. Thus, because obesity has both proliferative effects on the dynamics of oncogenesis and negative effects on protective mechanisms, it is expected that obese persons should be prone to malignant problems that are both frequent and highly variable in their level of aggression.

In contrast to obese persons, we predict that lean healthy persons are expected to be prone to malignant growths that are rare, but more likely to be aggressive—lean persons generally present unfavorable conditions for malignant progression and possess more protective mechanisms than obese persons. Together, these protective factors are expected to select for highly aggressive cells (i.e., that overwhelm defenses), whereas obese persons are expected to show more numerous but less aggressive neoplasia. While the global picture suggests that obesity could on average protect from developing aggressive cancers (Lennon et al., 2016), we suggest that this might be just an misinterpretation. The lack of aggressive and likely malignant neoplasia could explain the high incidence and multiplicity of cancer on the one hand, and higher survivorship on the other. Therefore, the true test to resolve the obesity paradox would be to consider the raw quantity, not the relative frequency of aggressive versus nonaggressive cancer cells between obese and normal weight persons. In accordance with our hypothesis, it has for example been demonstrated that renal cell cancers are often less aggressive in the obese (Albiges et al., 2016; Hakimi et al., 2013; Nishihara et al., 2015). Further studies are, however, necessary to fully determine why several obesity‐related cancers (see above) do not adhere to the obesity paradox, and hence deviate from the logic of our hypothesis. Similarly, further studies also need to explore the extent to which the enhanced survival in the obese is attributable to the reduced aggressivity of malignant cells and/or to the fact that those cells are more sensitive to treatments.

Another mechanism contributing to this apparent “paradox” may involve inhibitory effects of abundant nonaggressive neoplasia on those likely to become aggressive. That is, in obese patients the faster dynamics of oncogenesis together with lower defenses (see above) favor, at least sometimes, the early development of more benign tumors that exert negative effects on subsequent neoplasm formation—thus, emergence of aggressive tumors could be reduced. This could, for instance, be the case if aggressiveness in cancer is associated with the number of functional/driver mutations in a tumor (Maley et al., 2006), such that less aggressive tumors are more likely to appear first. This hypothesis is in fact an extrapolation of the phenomenon of “concomitant resistance” where the primary tumor prevents growth of metastasis (Benzekry, Gandolfi, & Hahnfeldt, 2014; Guba et al., 2001; Ruggiero et al., 2012).

Following the emergence of premalignant lesions, the survival of cancer clusters relies on increased production of angiogenesis stimulators, such as vascular endothelial growth factor (VEGF), that attract nutrient and oxygen providing blood vessels to the growing tumors (Bremnes, Camps, & Sirera, 2006; Carmeliet, 2005; Peterson et al., 2012). If two tumors induce production of angiogenesis stimulators, it is likely that eventually a threshold will be reached, such that the host would respond with increased production of angiogenesis inhibitors (e.g., platelet factor 4 [PF‐4], thrombospondin‐1 [TSP1], and endostatin), to counteract systemic increase in angiogenesis stimulators, which may be sufficient to restrict the growth of the smaller secondary tumors (Kareva, 2017). Interestingly, TSP1 is over‐expressed in obese patients (Varma et al., 2008), and this phenomenon may contribute to the obesity paradox. While more easily initiated, the TSP1 response in obese individuals may contribute to less aggressive and life‐threatening cancers. This scenario, which could explain the obesity paradox, also suggests that if the less aggressive tumors are surgically resected in obese patients, it could cause systemic decrease in the level of angiogenesis stimulators, which would be followed by decrease in the levels of angiogenesis inhibitors as the host's body restores homeostasis of angiogenesis regulators. This decrease could subsequently provide a “window of opportunity” for other, more aggressive, tumors to start growing (Demicheli, Retsky, Hrushesky, Baum, & Gukas, 2008). This is an additional reason why understanding whether or not an obesity paradox exists and its eventual causes are crucial. As suggested above, postulated mechanisms include angiogenesis, but immunity and even tyrosine isomers could play a role. For instance, Ruggiero et al. (2012) found in three murine models showing concomitant resistance (i.e., tumor‐bearing hosts being resistant to the growth of secondary tumor implants and metastasis) that both meta‐tyrosine and ortho‐tyrosine inhibited tumor growth, and even sometimes blocked metastasis. Beyond the obesity context, the reasoning presented in this paper could be extended to the population in general, whether obese or not. Indeed, by extension, it is expected that cancers in people with a healthy lifestyle should, all things being equal, be rarer but more aggressive than in persons with an unhealthy lifestyle. Further research should be conducted to investigate this issue and to determine the nature of the interactions between co‐occurring tumors during life.

## CONFLICT OF INTEREST

None declared.
